# Endometrial Immune Profiling: A Method to Design Personalized Care in Assisted Reproductive Medicine

**DOI:** 10.3389/fimmu.2020.01032

**Published:** 2020-06-04

**Authors:** Nathalie Lédée, Marie Petitbarat, Laura Prat-Ellenberg, Géraldine Dray, Guy N. Cassuto, Lucie Chevrier, Alaa Kazhalawi, Katia Vezmar, Gerard Chaouat

**Affiliations:** ^1^MatriceLAB Innove, Pépinière Paris Santé Cochin, Hôpital Cochin, Paris, France; ^2^Centre d'Assistance Médicale à la Procréation, Hôpital des Bluets, Paris, France; ^3^Laboratoire Drouot, Paris, France; ^4^INSERM U-976, St-Louis Hospital, Paris, France

**Keywords:** immunology, embryo implantation, endometrium, IVF, pregnancy

## Abstract

**Objective:** To assess the efficiency of the endometrial immune profiling as a method to design personalized care to enhance the pregnancy rate in a large heterogeneous infertile population. We hypothesized that some reproductive failures could be induced by a uterine immune dysregulation which could be identified and corrected with a targeted plan.

**Design:** Prospective cohort study.

**Setting:** Multicentric study.

**Intervention(s) and Main outcome measure(s):** One thousand and seven hundred thirty-eight infertile patients had an immune profiling on a timed endometrial biopsy between 2012 and 2018. This test documented the absence or the presence of an endometrial immune dysregulation and identified its type. In case of dysregulation, a targeted personalized plan was suggested to the treating clinician aiming to supply the anomaly. One year after the test, the clinician was contacted to provide the outcome of the subsequent embryo transfer with the applied suggested plan.

**Result(s):** After testing, 16.5% of the patients showed no endometrial immune dysregulation, 28% had a local immune under-activation, 45% had a local immune over-activation, and 10.5% had a mixed endometrial immune profile. In patients with a history of repeated implantation failures (RIF) or recurrent miscarriages (RM), the pregnancy rate was significantly higher if an endometrial dysregulation was found and the personalized plan applied, compared to the patients with an apparent balanced immune profile (respectively 37.7 and 56% vs. 26.9 and 24%, *p* < 0.001). In contrast, in good prognosis IVF (*in vitro* fertilization) subgroup and patients using donor eggs, this difference was not significant between dysregulated and balanced subgroups, but higher pregnancy rates were observed in absence of dysregulation. For patients with immune over-activation, pregnancy rates were significantly higher for patients who had a test of sensitivity, regarding the type of immunotherapy introduced, when compared to the ones who did not (51 vs. 39.9%, *p* = 0.012).

**Conclusion(s):** Local endometrial immunity appears to be a new and important parameter able to influence the prognosis of pregnancy. Targeted medical care in case of local immune dysregulation resulted in significantly higher pregnancy rates in RIF and RM patients.

## Introduction

Three demographic surveys published in the new millennium put the infertility figures in the many millions (ranging from 48.5 to 186 M) (1–3). Infertility is estimated to affect between 8 and 12% of reproductive-aged couples worldwide (4), with 9% currently cited as the probable global average (1). In 2010, of an estimated 11 million infertile Europeans (prevalence: 9%), half seek medical assistance and 22% receive fertility treatments. A half million assisted reproductive therapy (ART) cycles are performed annually in Europe (5) and expected to rise within the next decade as the prevalence of infertility climbs (due to maternal age and male infertility. Mathematical models suggest that 15% of patients enrolled in IVF cycles will have an history of repeated implantation failures and 1% of initiated pregnancies end up in recurrent miscarriages (6). The main brake for the success of assisted reproductive treatments remains the still low implantation rate of transferred embryos described by R. G. Edwards as “the last barrier” in reproductive medicine (7). Only 15 to 20% of day-3 embryos and 30% of day-5 embryos will effectively lead to a birth. In humans, indeed, most pregnancy losses occur before or during embryo implantation (8).

The endometrial immune profiling is a novel concept based on the analysis of the local immune reaction occurring in the endometrium at the time of the implantation window. The main hypothesis is that some failures (despite the production of good quality embryos) could be the consequence of uterine immune dysregulations, which could be anticipated and corrected with personalized therapies. Uterine immune profiling aims to counteract this high rate of embryo implantation failures through a better understanding of the uterine immune environment. Our final objective is to promote locally the mechanisms leading to an adequate immunological tolerance which has been shown to be essential for effective placentation.

In human, a crucial uterine immune reaction occurs, each cycle, in the mid-luteal phase independently from the simultaneous presence or absence of an embryo (9). The human endometrium prepares itself to be able to accept the embryo but only for a few days in each cycle. This period named as the implantation window is the period of uterine receptivity and occurs 5 to 9 days after ovulation.

During this specific period, a fundamental immune switch should occur locally to not only avoid the rejection of the semi-allogenic embryo but also to promote its growth and nutrition (10). At that time, important immune cells belonging to our adaptive immunity escape from the endometrium (as lymphocytes B, some Lymphocytes CD8). In the same time, immune cells belonging to the innate immunity (macrophage, uterine natural killer (uNK) cells, innate lymphoid cells- 1 and -3 and dendritic cells) invade the endometrium (11–13). The newly created immune environment plays a crucial role in embryo implantation (11). uNK cells differ from circulating NK cells by cytokines they produce and their low cytotoxic potential, by their phenotype and the repertory of activating and inhibiting receptors (14). To control the adaptive system, regulatory T cells, a subset of suppressor CD4(+) T cells, play a dominant role in the maintenance of immunological self-tolerance by preventing immune and autoimmune responses against self-antigens (15).

Previous authors documented by flow cytometry or immunohistochemistry abnormal immune cell mobilization or expression in patients with either RIF or RM history (13, 16–18) suggesting that endometrial immune local imbalance may contribute to implantation failures.

The immune profiling is based on RT-qPCR analysis of CD56 uNK levels, IL-15/Fn-14 ratios (Interleukin-15/Fibroblast growth factor-inducible molecule) and IL-18/TWEAK ratios (Interleukin-18/Tumor necrosis factor-like weak inducer of apoptosis), all factors known to be intimately involved in the differentiation of the secretory endometrium toward the receptive state.

To document the local Th-1/Th-2 balance, we chose to quantify the local expression of interleukin-18 because of its bivalence. IL-18 is a Th-2 angiogenic cytokine with an important demonstrated role in the destabilization of spatial arteries (19, 20), a crucial step to prepare the future invasion of the spiral arteries by the cytotrophoblast. However, a local over-expression of IL-18 switches its beneficial role to a deleterious one as IL-18 becomes Th-1 and promoter of local cytotoxicy (21). The level of local immune regulation which characterizes each individual will be also essential to promote or not a Th-1 deviation. Tumor necrosis factor-like weak inducer of apoptosis (TWEAK) and its receptor, fibroblast growth factor-inducible molecule (Fn14) are involved in preventing local cytotoxicity and counterbalancing the cytotoxic function of uNK cells (18, 21, 22). The maturity of uterine NK cells as well as their state of activation seems also essential (23). Uterine natural killer cells are not fully mature and should complete a process of maturation to gain effective functions (23). Interleukin-15 is the central cytokine for their recruitment and maturation in the endometrium (24). An over-expression of IL-15 is however able to activate all the local immune cells in a negative pathway. The ratio IL-15/Fn-14 is used in the immune profiling as a biomarker of the regulated state of maturation and activation of uNK cells. Moreover, we quantify the recruitment of CD56 positive cells (marker of uNK) initially by immunochemistry and currently by Real-Time PCR.

By documenting the local immune response expected during the period of uterine receptivity, we seek to detect imbalances which can be corrected to promote further embryo implantation.

Understanding the local uterine immune environment might be important to anticipate the future interplay between the endometrium and the embryo. Underactive immune cells may fail to create the necessary implantation reaction. Conversely, overactive immune cells may lead to a premature endometrial destruction and eventually to the rejection of the embryo. This unique immune reaction is essential for promoting embryo adhesion and its disruption is likely to impede implantation.

Initially developed in France for patients with a history of repeated implantation failures (RIF) (25, 26), we describe here the clinical results observed in 1,738 infertile patients and document its efficacy in clinical settings other than those previously studied as good prognosis infertile patients, patients with recurrent miscarriages or oocyte recipients. Between 2012 and 2018, based on the 1,738 documented immune profiles, suggestions of personalized care were proposed to the clinician, to be applied within the next 6 months or at the subsequent embryo transfer. The objective of personalized care is to restore the endometrial immune environment balance. In order to evaluate the efficacy of personalized care based on immune profiling, clinicians were contacted 1 year after the sample analysis to document the outcome of the first subsequent embryo transfer following the analysis, or within the next 6 months if no IVF treatment or embryo transfer was planned (in case of repeated miscarriages for spontaneous pregnancies).

## Materials and Methods

### Protocol Approval and Patient Consent

In 2011, the Institutional Review Board and the Ethical Committee of St. Louis Hospital approved the prospective follow-up of a cohort after immune profiling in order to document their outcome and a potential benefit (ref. 2011-A00994-37). All women provided written informed consent at the time of the endometrial collection, allowing to be contacted directly or through their physicians to collect the outcome at the first subsequent transfer following the uterine immune profiling. In 2014, we launched a randomized control study, the PRECONCEPTIO trial (NCT02262117). The PRECONCEPTIO trial evaluates the interest of the Pre-conceptional Endometrial Immune Profiling to Increase Birth Rates (NCT02262117) in good prognosis IVF patients. This randomized control trial (RCT) began to include patients in August 2016 and plan to randomize 400 dysregulated patients. Good prognosis infertile patients beginning their IVF treatment are included and have an endometrial immune profiling before their IVF. If a dysregulation is diagnosed, randomization allocates the choice between conventional vs. personalized IVF cycle. The outcome is the live birth rate at the first subsequent embryo transfer. 270 patients have been randomized so far. From October 2014 to August 2016, we began this RCT with the same inclusion criteria but the randomization allocated the choice between endometrial analysis vs. no endometrial analysis with the same outcome. We decided to give up this research design because in case of no analysis, we miss some crucial information as the presence or absence of dysregulation and its type which will be essential for further analysis. In the present trial, good prognosis IVF patient included were either randomized for endometrial analysis before August 2016 or personalized care after august 2016. The final results of the PRECONCEPTIO trial are expected in September 2022.

### Patients

The following information were for the patients undergoing endometrial biopsy: the age, the etiology of the infertility, the number of previous oocytes collections, the cumulated total number of embryos replaced, and the next treatment plan (fresh IVF with own eggs, Frozen embryo transfer FET, IVF with donor eggs IVF-OD, monitored natural cycles).

One thousand seven hundred and thirty-eight patients were included in this prospective cohort study. According to the data collected, patients were divided in different categories according to the clinical context (IVF/ICSI; oocyte donation, recurrent miscarriages) and to the number of attempts and the number of embryos previously replaced (beginning their course in IVF or IVF-OD vs. history of RIF with their own oocytes or with OD) (Table 1):

- 1,145 patients had a history of RIF, defined as the failure to get pregnant despite the cumulated transfers of at least 6 embryos over at least two oocytes collections for patients younger than 43 years old.
- 210 patients were infertile young patients (under 38 years old) with a normal ovarian reserve (AMH>1.5 ng/ml) evaluated before their first IVF cycle or after 1 IVF failure (good prognosis). These good prognosis infertile patients benefitted from the immune profiling either because they were referred for an exploration by their clinicians due to unexplained infertility, or because they were involved in the ongoing randomized control trial and were randomized for a personalization if a dysregulation was diagnosed (Preconception Trial).
- 91 patients were beginning their treatment in oocyte donation (abroad) and were evaluated either before their first embryo transfer or because they failed to get pregnant after only one embryo transfer (OD).
- 128 patients had a history of RIF in oocytes donation (more than 4 embryos transferred over two ET) with no pregnancy (RIF-OD).
- 164 patients had at least three consecutive repeated miscarriages (RM).

**Table 1:** Summarize of clinical data of patients included in this study.

**Clinical context**	**RIF**	**Good prognosis IVF**	**RM**	**OD**	**RIF-OD**
Number of patients	1,145	210	164	91	128
Mean age (years) [min–max]	35.7 [22.3–42.8]	33.2 [24.9–38.8]	35.7 [24.8–42.7]	39.45 [25.6–47]	39.9 [27–50]
Range of oocytes collection	3.6 [2–9]	0.86 [0–1]	0.23 [0–2]	–	–
Cumulated number of embryo transferred [min–max]	9 [6–35]	3.1 [0–6]	0.55 [0–3]	0.56 [0–3]	7.2 [4–25]
Number of miscarriages	0.55 [0–5]	0.21 [0–2]	3.87 [3–9]	0.61 [0–4]	0.69 [0-4]

*RIF, Repeated Implantation Miscarriages; IVF, In vitro Fertilization; RM, Recurrent Miscarriages; OD, Oocyte Donation*.

### Endometrial Biopsy: Collection and Analysis

From an endometrial biopsy collected by aspiration during the mid-luteal phase few months before assisted reproductive treatment, we quantified by quantitative Real-Time PCR five targeted biomarkers (IL-18, IL-15, TWEAK, Fn-14, and CD56) to document the immune endometrial environment in which the embryo will be transferred. A patent untitled “METHOD FOR INCREASING IMPLANTATION SUCCESS IN ASSISTED FERTILIZATION” describes the present invention as a method for determining a uterine receptivity profile in order to increase implantation success in assisted fertilization (PCT/EP2013/065355). In the patent, we defined the norms of expression for our biomarkers in a fertile cohort, and documented that an immune profile was reproducible from one cycle to another over a period of 6 months if no surgery or pregnancy occurred in the meantime.

Biopsies were collected by the referring clinicians during the mid-luteal phase, in a mock substituted cycle after 5 to 9 days of progesterone intake or in a monitored natural cycle. The endometrial fragment was gently aspirated by rotating a Pipelle de Cornier within the endometrial cavity. The Pipelle is the most studied biopsy device in the literature, it's a single use latex-free medical device for endometrium aspiration (27). The Pipelle content was divided into two parts: the first part was placed in 4% formaldehyde (QPath Formol 4% buffered, VWR Chemicals, Fontenay-sous-Bois, France) for endometrial datation (histological test to determine the phase of the cycle) and CD56 immuno-labeling, and the second part was placed in RNAlater stabilization solution for immunological analysis (MatriceLab Innove, France). The samples were sent at room temperature by postal services.

After confirmation of the mid-luteal phase by histological analysis, RNA was extracted and reverse-transcribed by RT-PCR. IL-15/Fn-14 and IL-18/TWEAK mRNA ratios were determined by quantitative RT-PCR with the Light Cycler 480 SYBR Green I Master mix (Roche Diagnostic). uNK cells recruitment was initially assessed using immunochemistry labeling positive CD56 cells and is currently assessed by Real time PCR (25, 26).

### Diagnosis of Endometrial Immune Profile

The local immune profile was defined according to the local balance of the mRNA expression of the two ratios IL-18/TWEAK, IL-15/Fn-14, and CD56 immunostaining or mRNA expression (25, 26).

Four types of endometrial immune profile were diagnosed:

- A BALANCED endometrial immune activation characterized by IL-18/TWEAK and IL-15/Fn-14 mRNA ratios and CD56+ cell count in the same range than previously defined in the fertile cohort.
- A LOW endometrial immune activation profile is characterized by low mRNA ratios for IL-15/Fn-14 (reflecting immature uNK cells) and/or IL-18/TWEAK or an absence of uNK recruitment.
- An OVER endometrial immune activation is characterized by high mRNA ratios of IL-18/TWEAK and/or IL-15/Fn-14 and/or a high CD56+ cell count.
- A MIXED endometrial immune profile is characterized by a high mRNA ratio of IL-18/TWEAK (excess of Th-1 cytokines) simultaneously with a low IL-15/Fn-14 ratio (reflecting immature NK).

### Suggestion of Personalized Treatment Plan

Based on the immune uterine profile, the clinician received 3 weeks after the endometrial collection the type of the local immune profile (no dysregulation, low immune activation, over immune activation, mixed profile) and a suggested personalized plan to supply the local imbalance if diagnosed (Table 2).

**Table 2:** Summary of the suggested protocols according to the immune profile documented.

**Suggestion of personalization/immune profile**	**No dysregulation**	**Low immune activation**	**Over immune activation**	**Mixed profile**
Endometrial scratching	No	Yes	No	Yes
Exposure to high concentration of Estrogens in the proliferative phase	No impact	No	Yes	Yes
Immunotherapy	No	No	Yes (therapy test)	Yes (therapy test)
Hormonal adaptation of the luteal phase	No	No	Yes	Yes
Luteal hCG supplementation	No	Yes	No	Yes
Exposure to seminal plasma	No impact	Yes	No	No

Suggestions were organized in six sections as follow.

#### Endometrial Scratching in the Mid-Luteal Phase of the Cycle Preceding the Transfer

Endometrial scratching was recommended in case of low IL-15/Fn-14 mRNA ratio interpreted in our hand as an immaturity of uNK cells. Endometrial scratching could enhance uNK cell maturation, which strongly depends on the adequate expression of IL-15 (28). Any type of local injuries (as endometrial biopsy) performed during the mid-luteal phase of the cycle activates and stimulates, at the next luteal phase, the subsequent expression of adhesion molecules and, interestingly, IL-15, via toll-like receptor pathways (29). The mechanism of action of endometrial scratching we want to trigger relies on the presence of innate immune cells. The positive effect of endometrial scratching is therefore only observed if performed in the mid-luteal phase and not observed if performed during the proliferative phase. IL-15 activates uNK cell maturation for women with a low IL-15/Fn-14 mRNA ratio.

#### Usage of High Level of Estrogens

High concentrations of estrogens (induced by ovarian hyper-stimulation during IVF cycle) has been described to down-regulate the endometrial IL-18 angiogenic expression (30–32). We therefore suggested avoiding such exposure if the IL-18 local expression was documented as low.

#### Immunotherapy

Adjunction of immunotherapy was proposed in over activated and mixed profiles either to decrease Th-1 cytokines or to control the recruitment and/or activation of uNK cells. As a first-line treatment, corticosteroid adjunction was recommended (33). In routine practice, we still lack precise indications for its use based on objective testing (34–37).

The rationale behind the prescription of corticosteroids in such immune profiles is based on the previous reports describing them to:

- decrease levels of Th-1 cytokines, NK cytotoxicity, and hyper-activation in lymphokine-activated killer cells (38).
- limit the consequences of IL-15 mRNA overexpression (39).
- modulate the Th1/Th2 balance when it is predominated by Th1 cytokines (40).

In case of resistance to corticosteroids, low molecular weight heparin was an option for their well-documented anti-complement effect (41, 42).

As a second line of treatment, we also evaluated the efficiency of intravenous slow perfusion of intralipids®. Previous authors reported that intravenous slow perfusion of diluted Intralipid® could be useful to control the hyper-activation of circulating NK cells and to regulate a Th-1 predominant cytokine balance (43, 44). We personally observed and reported a local reduction of Th-1 cytokines as well as a better control of uNK cells recruitment after intravenous perfusion of intralipids (45).

Intravenous intralipids perfusion was systematically performed under medical supervision at the hospital.

At this stage, regarding the choice of immunotherapy, only the normalization of the uterine immune profile under therapy could indicate the efficiency of any drug, since we are unable to predict the response to each type of therapy.

#### Hormonal Adaptation of the Luteal Phase

An adaptation of luteal support was recommended in over-activated profiles and mixed profiles.

Progesterone, beside its endocrine role, is a crucial mediator of the local immune tolerance required for a successful pregnancy.

Progesterone influences the maternal immune system through distinct pathways:

- Via the production of progesterone-induced blocking factor (PIBF), which inhibits NK cell activity (46) and leads to Th-2-dominant cytokine production by maternal lymphocytes (47).
- Via the induction of galectin-1, a progesterone-induced molecule, essential for inducing tolerogenic dendritic cells, which in return promotes *in vivo* expansion of IL-10 secreting Treg cells (48).

Hence, when a local immune over-activation was diagnosed, we recommended high daily vaginal doses of progesterone (1,200 mg) for its immunosuppressive properties. When the IL-18 expression was elevated, we also recommended oral estradiol supplementation (4 mg) to downregulate the local expression of IL-18 as previously described (30). The treatment would begin on the day of oocyte retrieval and be continued until 8 weeks after ET for pregnant women.

#### Supplementation of the Luteal Phase With Human Chorionic Gonadotrophin (hCG)

Previous authors demonstrated that hCG triggers both the proliferation and the maturation of uNK cells (49) and promotes local angiogenesis (50, 51). Physiologically produced by the embryo, hCG is directly involved in the local reaction through the induction of an adequate angiogenesis while controlling the activation of uNK cells at the maternal-fetal interface (52, 53). We recommended hCG supplementation during the mid-luteal phase in case of low CD56 recruitment or immaturity of uNK cells.

#### Sexual Intercourse After the ET

By inducing expression of pro-inflammatory cytokines and chemokines and the robust recruitment of immune cells, seminal plasma may have a positive role in preparing the endometrium for acceptance of implantation (54–56). We therefore recommended sexual intercourse in case of low immune activation but did not recommend exposure to seminal plasma in case of over-activated and mixed profiles.

### Prospective Collection of the Outcome

We collected the data over 5 different periods of time: ***2014*** for the biopsies analyzed in 2012 to 2013, ***2015*** for the biopsies collected in 2014, ***2016*** for the biopsies collected in 2015, ***2017*** for the biopsies collected in 2016, and ***2018*** for the biopsies analyzed in 2017 until July 2018.

The referring clinician confirmed whether personalization suggested was applied, the type of ART used (IVF, FET, monitored natural cycle or IUI for RM patients) and the outcome.

Outcome was classified in three categories: ongoing pregnancy (over 3 months of pregnancy), miscarriage, and no pregnancy.

### Statistical Analysis

We considered the outcome as reliable under the following conditions:

- If an embryo transfer was performed (RIF, good prognosis IVF, OD, RIF-OD, some RM): pregnancy occurred after the first embryo transfer (fresh or frozen) performed within the 9 months following the endometrial immune profiling.
- If no embryo transfer was performed (RM with spontaneous pregnancies): pregnancy occurred within the next 6 months following the endometrial immune profiling with a personalization applied through monitored natural cycles.

Pregnancy was defined by the presence of at least one embryo with a cardiac activity over 12 weeks of amenorrhea.

The outcome was analyzed according to the clinical context (good prognosis IVF, RIF, RM, OD, RIF-OD), considering the immune profile, dysregulated vs. balanced, and considering the different types of endometrial dysregulation (immune over-activation, immune under-activation, mixed profile).

ANOVA test with one or two level of variances was used. A *p*-value below 0.05 was considered as significant.

## Results

### Comparison of the Repartition of the Distinct Immune Profiles for Each Clinical Context

Regarding the entire cohort, 17% of infertile patients were diagnosed as having no immune dysregulation, 28% had an immune under-activation, 45% had an immune over-activation, 10% had a mixed profile.

As shown in Table 3, no significant differences (*p* = 0.20) were observed regarding the repartition between distinct types of immune profiles when considering the clinical context.

**Table 3:** Comparison of the repartition of the distinct immune profiles for each clinical context.

**Endometrial immune profiling**	**Before OD**	**Before IVF**	**RIF-OD**	**RIF**	**RM**	**Average repartition**
Number of patients	91	210	128	1145	164	–
No dysregulation	20.9% [19/91]	12.4% [26/210]	11.6% [15/128]	17.2% [197/1145]	19.5% [32/164]	289 (16.6%)
Immune under-activation	25.3% [23/91]	34.3% [72/210]	33.6% [43/128]	27.2% [311/1145]	23.2% [38/164]	487 (28%)
Immune over-activation	39.6% [36/91]	43.8% [92/210]	43.7% [56/128]	45.9% [525/1145]	45.1% [74/164]	783 (45.1%)
Mixed profile	14.3% [13/91]	9.5% [20/210]	10.9% [14/128]	9.8% [112/1145]	12.2% [20/164]	179 (10.3%)

*RIF, Repeated Implantation Failures; IVF, In vitro Fertilization; RM, Recurrent Miscarriages; OD, Oocyte Donation*.

Moreover, we did not observe differences regarding the repartition between dysregulated vs. balances profiles when considering the clinical context (*p* = 0.09).

### Outcomes in Presence or Absence of Endometrial Immune Dysregulation in Different Clinical Contexts (Table 4)

Pregnancy rates (PR) were affected by the clinical context and the presence of local immune dysregulation. Regarding the clinical context, the PR observed in the groups with good prognosis IVF and RM were significantly higher than the one observed in the RIF group (56 and 50%, respectively vs. 36%, *p* < 0.001) and not significantly higher than the one observed in OD (45% in OD and 42% in RIF-OD). Miscarriages were significantly higher for patients involved in oocytes donation (OD and RIF-OD) when compared to patients involved in IVF (good prognosis and RIF) using their own oocytes (*p* = 0.02).

**Table 4:** Outcome considering the presence or absence of dysregulation and clinical context.

**Clinical context**	**Global PR**	**Immune profile**	**Number of patients**	**Miscarriage rate**	**No pregnancy**	**Pregnancy rate (PR)**	***P*-value for the PR**
OD	45%	Dysregulated	72	11%	46%	44%	0.64
		Not dysregulated	19	16%	31.6%	52.6%	
RIF-OD	42%	Dysregulated	113	14.90%	43.80%	41.6%	0.56
		Not dysregulated	15	0%	53.3%	46.70%	
Good prognosis IVF	56%	Dysregulated	184	7.60%	38%	55%	0.28
		Not dysregulated	25	4%	36%	57.6%	
RIF	36%	Dysregulated	948	8.40%	53.8%	38.4%*	0.002
		Not dysregulated	201	7.50%	65.6%	26.90%	
RM	50%	Dysregulated	132	12%	32%	57.6%*	0.001
		Not dysregulated	33	24%*	52%	25%	

*PR, Pregnancy Rate; RIF, Repeated Implantation Miscarriages; IVF, In vitro Fertilization; RM, Recurrent Miscarriages; OD, Oocyte Donation*.

Regarding the local immune dysregulations, pregnancy rates were significantly higher for RIF and RM patients if a dysregulation was identified and the suggested personalization applied compared to non-dysregulated patients (respectively 38.4 and 57.6% vs. 26.9 and 25%, *p* < 0.002).

In contrast, in good prognosis IVF and OD sub-groups, no difference was observed between dysregulated vs. non-dysregulated patients, but the highest pregnancy rates were observed in absence of dysregulation.

Occurrence of a new miscarriage was higher and at the edge of significance (24 vs. 12%, *p* = 0.057) for RM patients with no dysregulation diagnosed.

### Outcome in Patients With Dysregulated Immune Profiles (Table 5)

No significant differences for either pregnancy or miscarriage rates were observed when considering the type of dysregulations diagnosed (immune over-activation, immune under-activation, mixed profile) and hence, the type of personalized care applied.

**Table 5:** Outcome in deregulated immune profiles.

**Clinical context**	**Immune endometrial profile**	***n***	**Ongoing Pregnancy rate**	**Miscarriages rate**
Good prognosis IVF	Over-activation	92	51%	8%
	Under-activation	72	64%	5%
	Mixed	20	45%	10%
RIF	Over-activation	525	40%	8%
	Under-activation	311	38%	8%
	Mixed	112	31%	7%
OD	Over-activation	36	44%	8%
	Under-activation	23	43%	13%
	Mixed	13	46%	15%
RIF-OD	Over-activation	56	41%	14%
	Under-activation	43	42%	14%
	Mixed	14	43%	21%
RM	Over-activation	74	55%	15%
	Under-activation	38	66%	10%
	Mixed	20	50%	5%

*RIF, Repeated Implantation Miscarriages; IVF, In vitro Fertilization; RM, Recurrent Miscarriages; OD, Oocyte Donation*.

### Over-immune Activation and Test of Sensitivity to Immunotherapy

For patients having a dysregulated endometrium with either an over-activation or a mixed profile, we recommended a test cycle under therapeutic to confirm the efficiency of the chosen immunotherapy.

To test the sensitivity to immunotherapy, the treatment was introduced as recommended on a new substituted cycle: introduction on day-3 of the cycle for the corticosteroids, on day-8 for the IV perfusion of Intralipids, concomitant with luteal support for the LMWH). The endometrial immune profiling was performed as usual in the mid luteal phase as previously described.

After the therapeutic test, the immunotherapy was classified as efficient in case of normalization of the profile, resistant if the immune imbalance was still present or worsened, partially responsive if the correction was incomplete. In case of partial response, the recommendation was to associate the used therapy with another one. In case of resistance, the clinician was advised to change the medication used.

99 patients were assessed under corticosteroids. This was efficient in 47% (47/99), resistant in 45% (45/99) and partially responsive in 8% (8/99).

46 patients were assessed after intravenous slow perfusion of Intralipids. This was efficient in 65% (30/46), resistant in 20% (9/46) and partially responsive in 20% (7/46).

5 patients were assessed under LMWH and corticosteroids and were all classified as responsive.

On 964 patients with either over-activation or mixed profile, 150 patients had a therapeutic test and 814 patients did not.

The ongoing pregnancy rate was significantly higher in patients with a therapeutic test compared to the ones without (51 vs. 39.9%. *p* = 0.012). The miscarriage rate was the same in both groups (9%).

### Endometrial Immune Dysregulation and Maternal Age

All the patients using their own oocytes or involved in OD were subdivided in three categories according to their age: under 35 years old, between 36 and 40 and over 40 years old. The distribution of the distinct immune profiles was the same in the three categories (*p* = 0.9) suggesting that the endometrial immune profiling is not influenced by maternal age or by the oocyte quality.

As oocyte quality depends on maternal age and highly impacts the outcome, we analyzed for the patients having IVF with their own oocytes, the impact of the maternal age and the presence of endometrial dysregulation on the outcome compared to patients having treatment with donor oocytes. For patient using their own oocytes, the pregnancy outcome was significantly affected by both the maternal age (*p* < 0.0001) and the presence of endometrial dysregulation (*p* = 0.001), in every category of age. Such effect was not observed for patients using donor oocytes (*p* = 0.49) (Table 6).

**Table 6:** Endometrial dysregulation and maternal age.

**Maternal age**	**Endometrial profile**	**Number of patients using their own oocytes for ART**	**Mean PR**	**Number of patients using OD**	**Mean PR in OD**
Less than 35 years old	Dysregulated	678	51%	51	55%
	Not dysregulated	132	40%	1	–
Between 36 and 39 years old	Dysregulated	423	37%	41	39%
	Not dysregulated	86	22%	7	71%
More than 40 years old	Dysregulated	163	24%	93	38%
	Not dysregulated	37	10%	26	46%

*PR, Pregnancy Rate; ART, Assisted Reproductive Therapy; OD, Oocyte Donation*.

## Discussion

Immune profiling in reproductive medicine is a new concept suggesting that the local endometrial immunity at the time of implantation may be rebalanced in order to positively influence the pregnancy outcome. One thousand seven hundred and thirty-eight infertile patients were prospectively followed over 5 years to evaluate the efficiency of this new diagnosis on their chance to get pregnant.

This extensive trial gave us new insights on the absence of variation of distribution of the immune profiles when considering the clinical context or the maternal age.

The absence of variation is an indirect demonstration that endometrial immune regulation is not a necessary condition for implantation. The embryo itself is able to promote the adhesion or to control the local activation of immune cells if necessary. However, if the embryo does not have the capacity to control the local immune dysregulations, personalized treatment may be important to promote an effective initial dialogue.

The absence of variation when considering the maternal age also suggests that the oocyte's quality and the endometrial immune local balance appear to be independent.

In the subgroups where the oocyte's quality was controlled (Good prognosis IVF or OD), the highest pregnancy rates were observed in non-dysregulated patients. For dysregulated patients, the key missing data is what would have been their pregnancy rate if no personalization had been applied. In 2016, we launched a randomized control trial in good prognosis IVF patients to cover this question. Five hundred patients are to be enrolled. If a dysregulation is diagnosed, patients are randomized for either a conventional IVF cycle or a personalized IVF cycle. Results are expected by the end of 2021. Such trial is difficult to realize in a RIF population, since the patients rarely accept to be randomized for conventional cycle if a dysregulation is diagnosed. This randomized control trial should determine when endometrial immune testing may be useful to increase the like hood of pregnancy in the normal course of infertility management.

In the large prospective trial presented, the patients with RIF and RM benefited from the immune profiling, independently from their age, in case of diagnosed endometrial dysregulation. The design of personalized care according to their immune profile significantly enhanced their chances of pregnancy. On the contrary, if no dysregulation was observed in a RIF context, the prognosis was highly dependent on the maternal age as no proposition could be suggested on the endometrial side.

In patients with RM, the absence of dysregulation was associated with the lowest PR and high risk of miscarriage. In this non-dysregulated sub-group, we may postulate that we missed the identification of the mechanism generating the miscarriage and that our biomarkers are not sufficient to identify the underlying problem. Interestingly, recent single cell sequencing studies have identified distinct dNK1 dNK2 and dNK3 populations that may differ in RIF and RM and require markers other than the pan dNK marker CD56 to identify potential changes (57, 58). In contrast, if a dysregulation was identified, the prognosis to get pregnant seems highly improved by the immune-based care personalization. Endometrial dysregulation was observed for 80% of the 164 RM patients included and may represent a new option for their personalized care, independently from assisted reproductive treatment as IVF. This approach needs to be evaluated through a randomized control trial in RM patients.

Results regarding the endometrial immune profiling in oocyte donation have never been reported before. The repartition of endometrial dysregulations in patients using donor oocytes was the same as the one observed in IVF and RM patients. The outcome however was lower than expected since oocyte quality was controlled *per-se* in oocyte donation. Independently from the presence or absence of endometrial dysregulation, the percentage of miscarriages observed was significantly higher for patients using donor oocytes than the one observed in patients involved in IVF using own oocytes (12 vs. 8%, *p* = 0.02). Moreover, personalization did not result in a reduction of the observed high rates of miscarriages. This suggests that other factors influence the implantation in OD. The precise monitoring of the luteal phase in these patients with no ovarian activity may be essential and should be evaluated prospectively. The absence of menstrual cycle may also influence the immune personalization. For example, when an endometrial scratching was recommended, some clinicians performed the procedure under monophasic pills which is classically prescribed for OD planning. As the principle requires an actual luteal phase to be effective, endometrial scratching in these conditions would not be effective. We also did not investigate if the luteal hCG supplementation was as effective in OD as it is observed in ovulatory women. It is therefore possible that some adaptations are necessary to increase the efficiency of the personalization for patients with no ovarian activity and ovarian cycles.

The main innovation suggests that one solution does not fit for all. Over time, all the meta-analysis evaluating the performance in IVF of either the endometrial scratching, the administration of corticosteroids or any type of immunotherapy were repeatedly negative (59–61). This is totally consistent with our data. Only 30% of any entire population would benefit from a principle, whatever the principle tested according to the observed immune profiles.

Regarding the type of immunotherapy applied to control an endometrial immune over-activation, we still do not have the knowledge to determine which drug would be optimal. Many distinct immune mechanisms may induce an immune over-activation (a Th-1/Th-2 imbalance, a hyper-activation of uNK in lymphokines-activated killer cells, an uncontrolled activation of the complement, an activation of Treg cells in Th-17 cells etc.). It is therefore understandable that a distinct principle may be required to control the endometrial environment in each case. Only a repeated test using the drug may however attest of its efficacy in the state of our knowledge. Pregnancy rates were significantly higher in patients who had a therapeutic when compared with the ones who did not.

To conclude, the endometrial local immunity appears as a new and important parameter able to influence the prognosis of pregnancy. In RIF and RM patients, the diagnosis of an endometrial immune dysregulation assorted with personalized care resulted in significantly higher pregnancy rates than observed in non-dysregulated patients. Quantification of the exact benefit when a dysregulation is diagnosed and a targeted personal therapy applied (vs. conventional care) is currently under evaluation in a randomized control trial among good prognosis IVF patients. Local endometrial immunity appears to be independent from the maternal age and clinical context.

## Data Availability Statement

The datasets generated for this study are available on request to the corresponding author.

## Ethics Statement

The studies involving human participants were reviewed and approved by the Institutional Review Board of St. Louis Hospital (2011-A00994-37) approved the prospective follow-up. The patients/participants provided their written informed consent to participate in this study.

## Author Contributions

NL conceived the research, interpreted the uterine immune profiles to personalize subsequent recommendations and wrote the manuscript. LC, AK, and KV performed the molecular analysis. LP-E, GD, GNC, GC, and MP corrected the manuscript and participated from the beginning to the establishment of the innovative procedure.

## Conflict of Interest

NL and MP initiated the start-up company MatriceLAB Innove. They work for the company, which also funded the study. They also hold a patent covering the endometrial immune assessment test and appended recommendations (PCT/EP2013/065355). The remaining authors declare that the research was conducted in the absence of any commercial or financial relationships that could be construed as a potential conflict of interest.

## Abbreviations

OD: Oocyte donation
RIF: Repeated implantation failures
RM: Recurrent miscarriages
IVF: *in vitro* fertilization
ART: assisted reproductive therapy
PR: Pregnancy rate.
